# Supplementary material for: Diagnostic Models Combining Clinical Information, Ultrasound and Biochemical Markers for Ovarian Cancer: Cochrane Systematic Review and Meta-Analysis
Source: Cancers (Basel). 2022 Jul 26;14(15):3621. doi: 10.3390/cancers14153621 (PMC9332683; doi:10.3390/cancers14153621)
Supplement: Supplementary file 1 [file cancers-14-03621-s001.zip › Supplementary File S1 Medline search strategies.pdf]

**<u>Supplementary File S1   SEARCH STRATEGIES MEDLINE APRIL 2015 AND JUNE 2019</u>**

**APRIL 2015**

**<u>1. ULTRASOUND</u>**

**Database:   MEDLINE (Ovid) 1946 to April Week 3 2015**

1    exp Ovarian Neoplasms/di

2    exp Adnexal Diseases/di

3    ((borderline or border line) adj4 ovar$).tw.

4    exp Fallopian Tube Neoplasms/di

5    exp Peritoneal Neoplasms/di

6    exp Pelvic Neoplasms/di

7    ((ovar$ or adnexal or fallopian or peritoneal$ or pelvic) adj3 (cancer$ or carcinoma$ or malignan$ or mass or masses or cyst or cysts or neoplas$ or tumour$ or tumor$)).tw.

8    ((epithelial or germ cell) adj5 ovar$).tw.

9    or/1-8

10    exp ultrasonography/

11    ultraso$.tw.

12    (transvagina$ adj2 sonogra$).tw.

13    or/10-12

14    9 and 13

15    limit 14 to (human and yr=2009-2015)

16    IOTA.tw.

17    International Ovarian Tumor Analysis.tw.

18    ((ovarian or epithelial or adnex$ or fallopian or peritoneal or pelvic) adj3 (model$ or regress$ or rule$ or score$ or algorithm$ or term$ or definition$ or measure$)).ti,ab.

19    or/16-18

20    9 and 19

21    limit 20 to human

22    15 or 21

## 2. OVARIAN CANCER BIOMARKERS

**Database: MEDLINE (Ovid) 1946 to April Week 3 2015**

1 exp Ovarian Neoplasms/di

2 exp Adnexal Diseases/di

3 ((ovar$ or adnexal or fallopian or peritoneal$ or pelvic) adj3 (cancer$ or carcinoma$ or malignan$ or mass or masses or cyst or cysts or neoplas$ or tumour$ or tumor$)).tw.

4 ((borderline or border line) adj4 ovar$).tw.

5 exp Fallopian Tube Neoplasms/di

6 exp Peritoneal Neoplasms/di

7 exp Pelvic Neoplasms/di

8 ((epithelial or germ cell) adj5 ovar$).tw.

9 or/1-8

10 exp Tumor Markers, Biological/

11 exp Biological Markers/

12 Proteomics/

13 Genetic Markers/

14 Metabolomics/

15 multiplex$.tw.

16 multivariate.tw.

17 (CA125 or CA-125 or HE4 or OVA 1 or OVA1 or HCG or LDH or AFP).mp. or CEA.tw. [

18 CA-125 Antigen/

19 Chorionic Gonadotropin/

20 L-Lactate Dehydrogenase/

21 alpha-Fetoproteins/

22 Carcinoembryonic Antigen/

23 or/10-22

24 9 and 23

25 limit 24 to (humans and yr="2009-2015")

**JUNE 2019**

**Database: Ovid MEDLINE(R) <1946 to June Week 4 2019>**

1 exp Ovarian Neoplasms/di (11075)

2 exp Adnexal Diseases/di (18488)

3 ((borderline or border line) adj4 ovar$).tw. (1933)

4 exp Fallopian Tube Neoplasms/di (476)

5 exp Peritoneal Neoplasms/di (2823)

6 exp Pelvic Neoplasms/di (1411)

7 ((ovar$ or adnexal or fallopian or peritoneal$ or pelvic) adj3 (cancer$ or carcinoma$ or malignan$ or mass or masses or cyst or cysts or neoplas$ or tumour$ or tumor$)).tw. (94961)

8 ((epithelial or germ cell) adj5 ovar$).tw. (13814)

9 or/1-8 (107343)

10 exp ovarian neoplasms/ (80285)

11 "Neoplasms, Glandular and Epithelial"/ (6565)

12 exp ovary/ (88997)

13 11 and 12 (302)

14 or/10-13 (165173)

15 9 or 14 (207720)

16 exp ultrasonography/ (417752)

17 ultraso$.tw. (298477)

18 (transvagina$ adj2 sonogra$).tw. (1898)

19 or/16-18 (540840)

20 9 and 19 (8473)

21 IOTA.tw. (1644)

22 International Ovarian Tumor Analysis.tw. (78)

23 ((ovarian or epithelial or adnex$ or fallopian or peritoneal or pelvic) adj3 (model$ or regress$ or rule$ or score$ or algorithm$ or term$ or definition$ or measure$)).ti,ab. (13433)

24 or/21-23 (15075)

25 19 and 24 (965)

26 exp Tumor Markers, Biological/ (245215)

27 exp Biological Markers/ (696607)

28 Proteomics/ (47440)

29 Genetic Markers/ (54266)

30 Metabolomics/ (13222)

31 multiplex$.tw. (38220)

32 multivariate.tw. (261210)

33 (CA125 or CA-125 or HE4 or OVA 1 or OVA1 or HCG or LDH or AFP).mp. or CEA.tw. [mp=title, abstract, original title, name of substance word, subject heading word, floating sub-heading word, keyword heading word, organism supplementary concept word, protocol supplementary concept word, rare disease supplementary concept word, unique identifier, synonyms] (85477)

34 CA-125 Antigen/ (4595)

35 Chorionic Gonadotropin/ (27724)

36 L-Lactate Dehydrogenase/ (40512)

37 alpha-Fetoproteins/ (15565)

38 Carcinoembryonic Antigen/ (15115)

39 or/26-38 (1100948)

40 exp "Signs and Symptoms"/ (1982694)

41 symptom$.ti,ab. (918726)

42 exp early diagnosis/ or exp Diagnosis/ (8189592)

43 exp "Early Detection of Cancer"/ (21502)

44 (early adj (sign$ or symptom$)).tw. (10387)

45 (abdom$ adj3 (pressure or pain$ or swelling$ or hard)).tw. (51786)

46 (bowel irregularit$ or bloat$ or fullness or satiet$ or gastro$).tw. (325941)

47 (fatigue or weight loss$ or weight gain$ or constipat$ or diarrhoea or diarrhea or gas).tw. (470228)

48 (nausea$ or indigestion).tw. (50305)

49 ((loss or lack) adj3 (energ$ or appetite$)).tw. (8255)

50 (urin$ adj3 (frequenc$ or urgenc$)).tw. (3991)

51 ((leg$ or ankle$) adj2 (swell$ or swollen)).tw. (1150)

52 ((abnormal or irregular or postmenopausal) adj1 vaginal adj (bleed$ or discharge$)).tw. (969)

53 (pelvic discomfort$ or pelvic pain$ or chest pain$ or respirator$ difficult$ or lower back pain$).tw. (37099)

54 or/40-53 (9815896)

55 (index or risk$ or score$ or scoring or checklist$ or rule$ or indices or tool$ or instrument$ or survey$ or questionnaire$ or interview$).tw. (4088951)

56 (LR2 or RMI or ROMA or ADNEX).mp. [mp=title, abstract, original title, name of substance word, subject heading word, floating sub-heading word, keyword heading word, organism supplementary concept word, protocol supplementary concept word, rare disease supplementary concept word, unique identifier, synonyms] (3291)

57 54 and 55 (2028010)

58 19 or 24 or 39 or 56 or 57 (3311230)

59 9 or 14 (207720)

60 58 and 59 (56137)

61 limit 60 to (humans and yr="2015 - 2019") (8770)

62 57 and 59 (17916)

63 61 not 54 (1928)

64 19 or 24 or 39 or 56 (1622241)

65 15 and 64 (45250)

66 limit 65 to (humans and yr="2015 - 2019") (6445)

67 57 or 64 (3311230)

68 59 and 67 (56137)

69 limit 68 to (humans and yr="2015 - 2019") (8770)
