# Supplementary material for: Diagnostic Models Combining Clinical Information, Ultrasound and Biochemical Markers for Ovarian Cancer: Cochrane Systematic Review and Meta-Analysis
Source: Cancers (Basel). 2022 Jul 26;14(15):3621. doi: 10.3390/cancers14153621 (PMC9332683; doi:10.3390/cancers14153621)
Supplement: Supplementary file 1 [file cancers-14-03621-s001.zip › Supplementary File S2 illustrating application of test accuracy metrics.pdf]

Supplemenatry file S2 illustrating application of test accuracy metrics

Pre - Menopausal Women

Prevalence of Ovarian Cancer in the population *(number of women who actually have Ovarian Cancer)*	Test result	Actual diagnosis

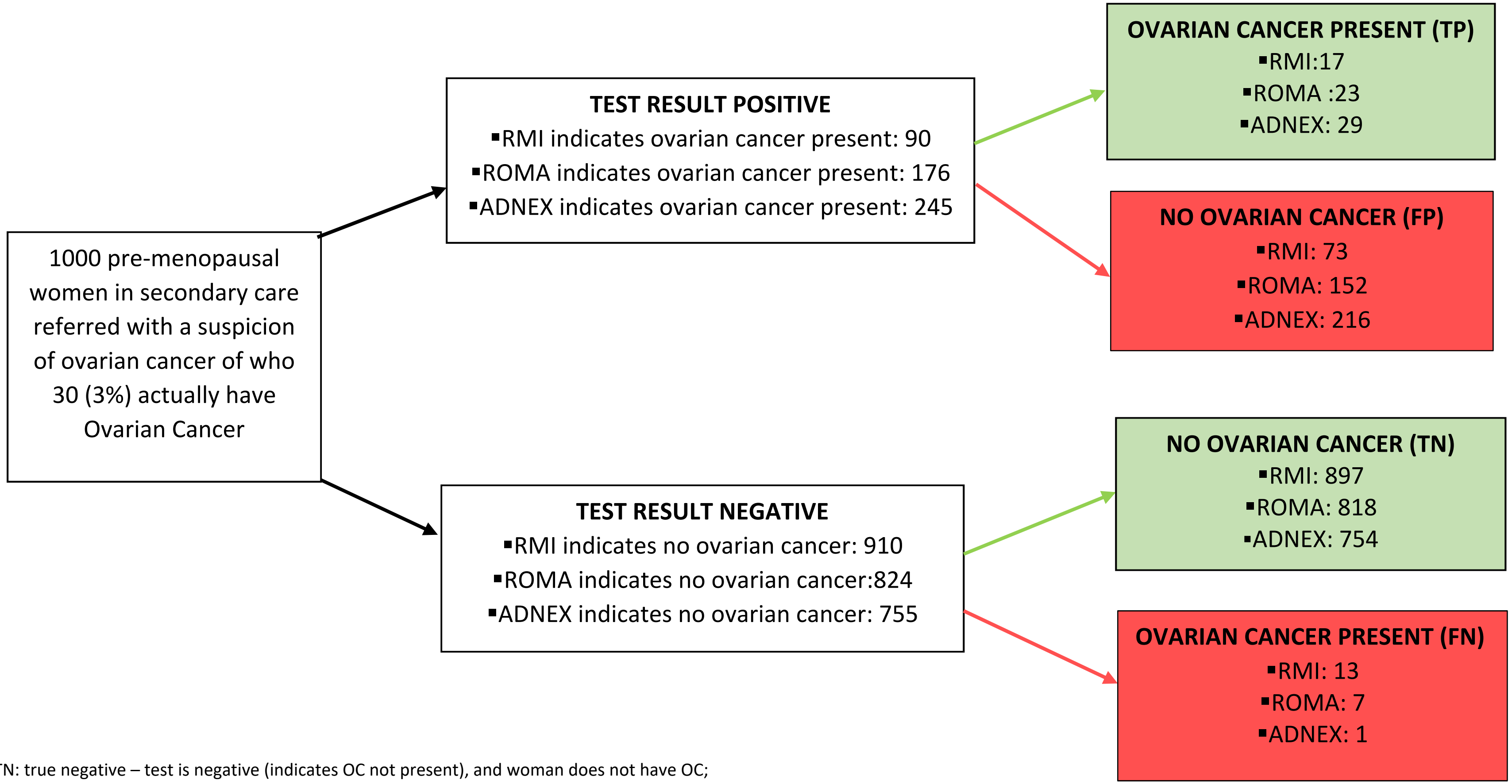

TN: true negative – test is negative (indicates OC not present), and woman does not have OC;
FN: false negative – test is negative (indicates OC not present), but woman has OC;

TP: true positive – test is positive (indicates OC is present), and woman has OC;
FP: false positive – test is positive (indicates OC is present), but woman does not have OC

Post - Menopausal Women

**Prevalence of Ovarian Cancer in the population** ***(number of women who actually have Ovarian Cancer)***	**Test result**	**Actual diagnosis**

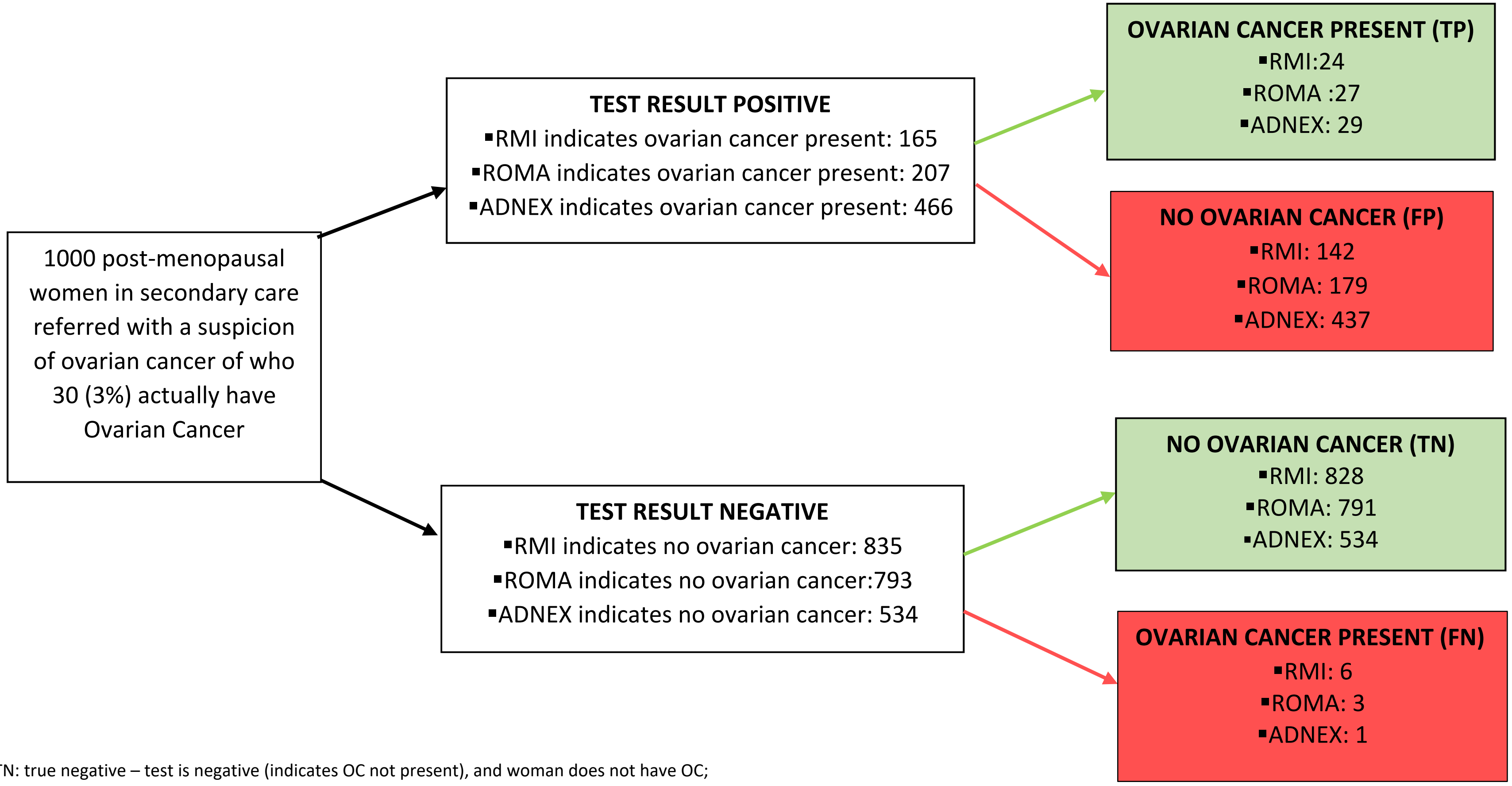

TN: true negative – test is negative (indicates OC not present), and woman does not have OC;
FN: false negative – test is negative (indicates OC not present), but woman has OC;
TP: true positive – test is positive (indicates OC is present), and woman has OC;
FP: false positive – test is positive (indicates OC is present), but woman does not have OC
