# Supplementary material for: Diagnostic Models Combining Clinical Information, Ultrasound and Biochemical Markers for Ovarian Cancer: Cochrane Systematic Review and Meta-Analysis
Source: Cancers (Basel). 2022 Jul 26;14(15):3621. doi: 10.3390/cancers14153621 (PMC9332683; doi:10.3390/cancers14153621)
Supplement: Supplementary file 1 [file cancers-14-03621-s001.zip › Supplementary File S3 Complete Summary Characteristics included studies. docx.pdf]

Supplementary File S3: Complete Summary Characteristics included studies

Study characteristics of included studies : RMI 1

Author Year Country	Setting	Participants characteristics	Index test threshold
**Abdalla 2017**<br>**Poland**	**Study criteria:** Women scheduled to undergo surgery for adnexal tumours<br>**Clinical setting:** Mixed<br>**Prior tests:** US assessment of adnexal mass and measurement of tumour markers CA125 and HE4 within 5 days before surgical intervention<br>**Exclusions:** Presence of fibroids > 5 cm were excluded<br>**Centre:** Single	**N:** 312<br>**Postmen n (%):** 117 (37)<br>**Ovarian cancer n (%):** 45 (15)<br>**Borderline n (%):** 7 (2)<br>**Age:** range 18-85<br>**Separated by menopausal status:** Yes	**Threshold:** 200<br>**Pre-specified:** Yes
**Al Musalhli 2016**<br>**Oman**	**Study criteria:** Patients with an ovarian mass<br>**Clinical setting:** Mixed<br>**Prior tests:** Unclear but assume US<br>**Exclusions:** None reported<br>**Centre:** Single	**N:** 213<br>**Postmen n (%):** 51 (24)<br>**Ovarian cancer n (%):** 48 (23)<br>**Borderline n (%):** 7 (3)<br>**Age:** Not reported<br>**Separated by menopausal status:** Yes	**Threshold:** 200<br>**Pre-specified:** Yes
**Anton 2012**<br>**Brazil**	**Study criteria:** Women referred with pelvic mass diagnosed by US, CT or MRI with signs of carcinomatosis undergoing surgery or image-guided biopsy.<br>**Clinical setting:** Secondary care<br>**Prior tests:** Unclear<br>**Exclusions:** None reported<br>**Centre:** Single	**N:** 120<br>**Postmen n (%):** 73 (60)<br>**Ovarian cancer n (%):** 30 (25)<br>**Borderline n (%):** 17 (14)<br>**Age mean:**<br>-Malignant: 54.7<br>-Borderline: 56.4<br>-Benign: 50.7<br>**Separated by menopausal status:** Yes	**Threshold:** 200<br>**Pre-specified:** Yes
**Ertas 2016**<br>**Turkey**	**Study criteria:** Women with adnexal masses that underwent surgery<br>**Clinical setting:** Tertiary<br>**Prior tests:** Unclear<br>**Exclusions:** None reported<br>**Centre:** Single centre	**N:** 408<br>**Postmen n (%):** 117 (71.4)<br>**Ovarian cancer n (%):** 55 (13)<br>**Borderline n (%):** 12 (3)<br>**Mean age (+/-SD):**<br>-Benign: 40.8 (13.8)<br>-Malignant: 54.4 (13.6)<br>**Separated by menopausal status:** Yes	**Threshold:** 200<br>**Pre-specified:** Yes

**Irshad 2013**<br>**Pakistan**	**Study criteria:** Unclear (ovarian masses)<br>**Clinical setting:** Secondary<br>**Prior test:** Unclear<br>**Exclusions:** Unclear<br>**Centre:** Single centre	**N**: 36<br>**Postmen n (%):** 36 (100)<br>**Ovarian cancer n (%)**: 24 (37)<br>**Borderline n (%):** Not reported<br>**Age mean:** 58<br>**Separated by menopausal status:** Yes	**Thresholds:** 250<br>**Pre-specified:** Yes
**Krascsenitis 2016**<br>**Hungary**	**Study criteria:** Women diagnosed with an ovarian tumour of unknown significance admitted for surgery<br>**Clinical setting:** Tertiary<br>**Prior tests:** Not reported<br>**Exclusions:** None reported<br>**Centre:** Single	**N:**162<br>**Postmen n (%):** 102 (63)<br>**Ovarian cancer n (%):** 34 (21)<br>**Borderline n (%):** 11 (7)<br>**Mean age (+/-SD):** 55<br>**Separated by menopausal status:** Yes	**Threshold:** 200<br>**Pre-specified:** Yes
**Liest 2019**<br>**Sweden**	**Study criteria:** Women with a pelvic mass of probable ovarian origin and scheduled for surgery<br>**Clinical setting:** Tertiary<br>**Prior tests:** Preoperative US<br>**Exclusions:** None reported<br>**Centre:** Multicentre	**N:** 784<br>**Postmen n (%):** 117 (81)<br>**Ovarian cancer n (%):** 144 (18) (include borderline)<br>**Borderline n (%):** Not reported<br>**Age:** Not reported<br>**Separated by menopausal status:** Yes	**Threshold:** 200<br>**Pre-specified:** Yes
**Lycke 2018**<br>**Sweden**	**Study criteria:** Women planned for a surgical procedure for a symptomatic/suspected malignant ovarian cyst or pelvic tumour<br>**Clinical setting:** Mixed<br>**Prior tests:** Unclear but assume history and examination, and US from patient selection<br>**Exclusions:** None reported<br>**Centre:** Multicentre	**N:** 638<br>**Postmen n (%):** 348 (55)<br>**Ovarian cancer n (%):** 162 (25)<br>**Borderline n (%):** 31 (5)<br>**Mean age (+/-SD):**<br>-Benign 50.76<br>-BOT: 55.58<br>-EOC: 62.67<br>**Separated by menopausal status:** Yes	**Threshold:** 200<br>**Pre-specified:** Yes
**Manegold Brauer 2016**<br>**Switzerland**	**Study criteria:** Women who had had an US examination for an adnexal mass with histology and CA125 results available<br>**Clinical setting:** Secondary<br>**Prior tests:** Not reported<br>**Exclusions:** None reported<br>**Centre:** Single centre	**N:** 1108<br>**Postmen n (%):** 478 (43)<br>**Ovarian cancer n (%):** 118 (11)<br>**Borderline n (%):** 33 (3)<br>**Mean age (+/-SD):** Median 48<br>**Separated by menopausal status:** Yes	**Threshold:** 200<br>**Pre-specified:** Yes

**Meys 2017**<br>**Netherlands**	**Study criteria:** Women with adnexal pathology<br>**Clinical setting:** Tertiary<br>**Prior tests:** Not reported<br>**Exclusions:** None reported<br>**Centre:** Single	**N:** 326<br>**Postmen n (%):** 198 (61)<br>**Ovarian cancer n (%):** 115 (35)<br>**Borderline n (%):** 27 (8)<br>**Median age (IQR):**<br>-Benign 53.2 (16.1 to 87.2)<br>-Malignant 67.7 (32.3 to 87)<br>**Separated by menopausal status:** Yes	**Threshold:** 200<br>**Pre-specified:** Yes
**Niemi 2017**<br>**Finland**	**Study criteria:** Women over 50 years of age presenting with an abnormal adnexal mass(es)<br>**Clinical setting:** Tertiary<br>**Prior tests:** Not reported<br>**Exclusions:** Overtly benign or malignant looking tumours like unilocular simple ovarian cysts and tumours associated with marked ascites (depth of the greatest pool over 10 cm) were excluded<br>**Centre:** Single	**N:** 98<br>**Postmen n (%):** 98 (100)<br>**Ovarian cancer n (%):** 23 (23)<br>**Borderline n (%):** 7 (7)<br>**Median age:** 61 (range 50 to 84)<br>**Separated by menopausal status:** Only post-menopausal included	**Threshold:** 200<br>**Pre-specified:** Yes
**Nikolova 2016**<br>**Macedonia**	**Study criteria:** Premenopausal women to have an ultrasonography scan confirming an ovarian cyst/mass and to undergo surgery<br>**Clinical setting:** Tertiary<br>**Prior test:** Unclear<br>**Exclusions:** Post-menopausal women excluded<br>**Centre:** Single	**N:** 105 (analysed)<br>**Postmen n (%):** 0<br>**Ovarian cancer n (%):** 11 (10%)<br>**Borderline n (%):** Not reported<br>**Mean age (+/-SD):**<br>OC: 42.46 (8.21)<br>Benign: 36.90 (10.12)<br>**Separated by menopausal status:** Only premenopausal women included	**Threshold:** 250<br>**Pre-specified:** Yes
**Radosa 2011**<br>**Germany**	**Study criteria:** women with adnexal mass who subsequently underwent surgery were selected<br>**Clinical setting:** Tertiary<br>**Prior test:** Unclear<br>**Exclusions:** Nil<br>**Centre:** Single centre	**N:** 442<br>**Postmen n (%):** 141 (32)<br>**Ovarian cancer n (%)**: 79<br>**Borderline n (%):** 19<br>**Age mean:** 43.3<br>**Separated by menopausal status:** Yes	**Thresholds:** 200<br>**Pre-specified:** Yes

**Richards 2015 Australia**	**Study criteria:** Women who were undergoing surgery for a complex pelvic mass, presumed to be arising from the ovary<br>**Clinical setting:** Mixed<br>**Prior tests:** Unclear<br>**Exclusions:** None reported<br>**Centre:** Single	**N:** 50<br>**Postmen n (%):** 29 (58)<br>**Ovarian cancer n (%):** 16 (32)<br>**Borderline n (%):** 4 (8)<br>**Median age:** 60<br>**Separated by menopausal status:** Yes	**Thresholds:** 200<br>**Pre-specified:** Yes
**Sayasneh 2013a UK**	**Study criteria:** Women presenting with adnexal mass and undergoing surgery within 120 days after examination<br>**Clinical setting:** Mixed<br>**Prior test:** Unclear<br>**Exclusions:** Nil<br>**Centre:** Multi centre	**N:** 255<br>**Postmen n (%):** 117 (46)<br>**Ovarian cancer n (%)**: 48 (19)<br>**Borderline n (%):** 18 (7)<br>**Age mean:** 46<br>**Separated by menopausal status:** Yes	**Thresholds:** 200<br>**Pre-specified:** Yes
**Terzic 2013 Serbia**	**Study criteria:** Women treated for adnexal tumours<br>**Clinical setting:** Secondary<br>**Prior test:** Unclear<br>**Exclusions:** Nil<br>**Centre:** Single centre	**N:** 689<br>**Postmen n (%):** 138 (20)<br>**Ovarian cancer n (%)**: 112 (16)<br>**Borderline n (%):** 33 (5)<br>**Age mean:**<br>- Benign: 42.8<br>- Borderline: 53.6<br>- Malignant: 57.25<br>**Separated by menopausal status:** Yes	**Thresholds:** 250<br>**Pre-specified:** Yes
**Testa 2014 European countries**	**Study criteria:** Women presenting with adnexal mass and undergoing TVS by one of the principal investigators and surgery within 120 days after examination<br>**Clinical setting:** Mixed<br>**Prior test:** Unclear<br>**Exclusions:** Nil<br>**Centre:** Single centre	**N:** 2403<br>**Postmen n (%):** 1049 (44)<br>**Ovarian cancer n (%)**: 701 (29)<br>**Borderline n (%):** 153 (6)<br>**Age mean**: Not reported<br>**Separated by menopausal status:** Yes	**Thresholds:** 200<br>**Pre-specified:** Yes

**Van den Akker 2016 Netherlands**	**Study criteria:** Women admitted for surgical treatment of an ovarian mass with unknown histology<br>**Clinical setting:** Mixed<br>**Prior tests:** Not reported<br>**Exclusions:** No such inappropriate exclusion; Women in whom clear evidence of malignancy was found before or during the surgical procedure (e.g. pleural effusions and evidence of distal organ involvement) were excluded<br>**Centre:** Multicentre	**N:** 670<br>**Postmen n (%):** 390 (58)<br>**Ovarian cancer n (%):** 93 (14)<br>**Borderline n (%):** 46 (6)<br>**Median age:** 54<br>**Separated by menopausal status:** Yes	**Threshold:** 200<br>**Pre-specified:** Yes
**Van Gorp (IOTA) 2012 Belgium**	**Study criteria:** Women with a pelvic mass, scheduled for surgery<br>**Clinical setting:** Secondary<br>**Prior test:** Unclear<br>**Exclusions:** Nil<br>**Centre:** Single centre	**N:** 374<br>**Postmen n (%):**196 (52)<br>**Ovarian cancer n (%)**: 94 (25)<br>**Borderline n (%):** 31 (8)<br>**Age mean:**<br>- Benign: 46.2<br>- Malignant: 57.7<br>**Separated by menopausal status:** Yes	**Thresholds:** 200<br>**Pre-specified:** Yes
**Vural 2016 Turkey**	**Study criteria:** Post menopausal women with adnexal masses who underwent surgery<br>**Clinical setting:** Tertiary<br>**Prior tests:** Not reported<br>**Exclusions:** Pre-menopausal excluded<br>**Centre:** Single	**N:** 139<br>**Postmen n (%):** 139 (100)<br>**Ovarian cancer n (%):** 44 (32)<br>**Borderline n (%):** 8 (6)<br>**Mean age (+/-SD):** 61.1 (8.9) (range 42 to 87)<br>**Separated by menopausal status:** Only post-menopausal	**Threshold:** 200<br>**Pre-specified:** Yes

Notes to table: Clinical Setting: Secondary care: dedicated gynaecologist in a general hospital; Tertiary care – gynaecological oncology centre.

Study Characteristics included studies - ROMA

Author Year	Setting	Participant characteristics	Index test threshold*
**Al Musalhli 2016 Oman**	**Study criteria:** Patients with an ovarian mass<br>**Clinical setting:** Mixed<br>**Prior tests:** Unclear but assume US<br>**Exclusions:** None reported	**N:** 213<br>**Postmen n (%):** 51 (24)<br>**Ovarian cancer n (%):** 48 (23)<br>**Borderline n (%):** 7 (3)	**Threshold:**<br>-Premen: 13.1<br>-Postmen: 27.7<br>**Pre-specified:** Yes

	**Centre:** Single	**Age:** Not reported<br>**Separated by menopausal status:** Yes	
**Anton 2012**<br>**Brazil**	**Study criteria:** Women presenting with signs of carcinomatosis with a pelvic mass diagnosed by US, CT or MRI undergoing surgery or image-guided biopsy.<br>**Clinical setting:** Secondary care<br>**Prior tests:** Not reported<br>**Exclusions**: None reported<br>**Centre:** Single	**N:** 120<br>**Postmen n (%):** 73 (60.8%)<br>**Ovarian cancer n (%):** 30 (25%)<br>**Borderline n (%):** 17 (14%)<br>**Age mean:**<br>-Malignant: 54.7<br>-Borderline: 56.4<br>-Benign: 50.7<br>**Separated by menopausal status:** Yes	**Thresholds:**<br>-Premen 13.1;<br>-Postmen 27.7<br>**Pre-specified:**<br>Yes
**Bandiera 2011**<br>**USA**	**Study criteria:** Not reported<br>**Clinical setting:** Tertiary care<br>**Prior tests:** Not reported<br>**Exclusions:** Non Epithelial Ovarian Cancer excluded<br>**Centre:** Single	**N:** 278<br>**Postmen n (%):** 183 (65.8)<br>**Ovarian cancer n (%):** 113 (41)<br>**Borderline n (%):** Not reported<br>**Mean age:**<br>Pre menopausal:<br>-Malignant: 44.7<br>-Benign: 41.5<br>Post menopausal:<br>-Malignant: 66.3<br>-Benign: 64.0<br>**Separated by menopausal status:** Yes	**Thresholds:**<br>-Premen 7.4;<br>-Postmen 25.3<br>**Pre-specified:** Yes
**Chan 2013**<br>**Countries in Asia-Pacific region**	**Study criteria:** Women over 18 years diagnosed with adnexal mass diagnosed by any Imaging method (US, CT or MRI)<br>**Clinical setting:** Unclear<br>**Prior test:** Unclear<br>**Exclusions:** Nil<br>**Centre:** Multicentre	**N:** 414<br>**Postmen n (%):** 26 (108)<br>**Ovarian cancer n (%):** 74 (18)<br>**Borderline n (%):** 16 (4)<br>**Age mean:** Not reported<br>**Separated by menopausal status:** Yes	**Thresholds:**<br>-Premen 7.4;<br>-Postmen 25.3<br>**Pre-specified:** Yes

**Chen 2015 China**	**Study criteria:** Women with pelvic masses scheduled for surgery<br>**Clinical setting:** Unclear<br>**Prior test:** Unclear<br>**Exclusions:** Nil<br>**Centre:** Single	**N:** 130<br>**Postmen n (%):** 62 (48)<br>**Ovarian cancer n (%):** 60 (46)<br>**Borderline n (%):** Not reported<br>**Age mean:**<br>- Benign: 34<br>- Malignant: 53<br>**Separated by menopausal status:** Yes	**Thresholds:**<br>- Premen 11.4<br>-Postmen 29.9<br>**Pre-specified:** Yes
**Chen 2014 China**	**Study criteria:** Women with EOC and Benign lesions<br>**Clinical setting:** Tertiary<br>**Prior test:** Unclear<br>**Exclusions:** Women with non EOC excluded<br>**Centre:** Single	**N:** 192<br>**Postmen n (%):** 84 (44)<br>**Ovarian cancer n (%):** 123 (64)<br>**Borderline n (%):** Not reported<br>**Age mean:** not reported<br>**Separated by menopausal status:** Yes	**Thresholds:**<br>- Premen 12.2;<br>- Postmen 25.8<br>**Pre-specified:** Yes
**Chudeka-Glaz 2016 Poland**	**Study criteria:** Consecutive women who attended the hospital presenting with suspected ovarian cancer (ovarian tumour, ovarian cyst, or ascites).<br>**Clinical setting:** Tertiary<br>**Prior test:** Not reported<br>**Exclusions:** None reported<br>**Centre:** Single	**N:** 413<br>**Postmen n (%):** 251 (61)<br>**Ovarian cancer n (%):** 162 (39)<br>**Borderline n (%):** Not reported<br>**Age median:**<br>-Benign: 35<br>-Malignant: 59.7<br>**Separated by menopausal status:** Yes	**a) ROMA**<br>**Thresholds:**<br>- Premen 14.1<br>- Postmen 25<br>**Pre-specified:** Yes<br><br>**b) ROMA-P**<br>**Thresholds:** Determined by age group in both pre- and post- menopausal; Age group includes:<20 years old, 21 to 30 years old, 31 to 40 years old, 41 to 50 years old, 51 to 60 years old, 61 to 70 years old, 71 to 80 years old, and >80 years old.<br><br>**Pre-specified:** No
**Cradic 2018 USA**	**Study criteria:** Women with EOC or benign ovarian lesions<br>**Clinical setting:** Tertiary	**N:** 207<br>**Postmen n (%):** 93 (45)	**Thresholds:**<br>-Premen 11.4

	**Prior test:** Not reported<br>**Exclusions:** Not reported<br>**Centre:** Single	**Ovarian cancer n (%):** 76 (37) (EOC)<br>**Borderline n (%):** Not reported<br>**Age mean:** Not reported<br>**Separated by menopausal status:** Yes	-Postmen 29.9<br>**Pre-specified:** Yes
**Dikeman 2015 Turkey**	**Study criteria:** Women were 'preoperative'<br>**Clinical setting:** Unclear<br>**Prior test:** Unclear<br>**Exclusions:** None reported<br>**Centre:** Unclear	**N:** 143<br>**Postmen n (%):** 46 (32)<br>**Ovarian cancer n (%):** 47 (33)<br>**Borderline n (%):** Not reported<br>**Age mean:**<br>-Benign: Mean 42 (SD 10)<br>-Malignant: 56 (SD14)<br>**Separated by menopausal status:** Yes	**Thresholds:**<br>-Premen 13.1<br>- Postmen 27.7<br>**Pre-specified:** Yes
**Farzaneh 2014 Iran**	**Study criteria:** Women with adnexal mass undergoing surgery and having attained menarche 12 months before presenting with adnexal mass<br>**Clinical setting:** Secondary<br>**Prior test:** Unclear<br>**Exclusions:** Excluded non EOC **Centre:** Single	**N:** 99<br>**Postmen n (%):** 31 (31)<br>**Ovarian cancer n (%):** 43 (43)<br>**Borderline n (%):** Not reported<br>**Age mean:**<br>- Benign: 39<br>-Malignant (EOC): 51<br>**Separated by menopausal status:** Yes	**Thresholds:**<br>- Premen 11.5;<br>- Postmen 25.5<br>**Pre-specified:** Yes
**Grenache 2015 USA**	**Study criteria:** Women with abnormal adnexal mass detected on physical examination and Imaging Included Ultrasound, CT or MRI) followed by surgery<br>**Clinical setting:** Unclear<br>**Prior test:** Unclear<br>**Exclusions:** Unclear<br>**Centre:** Multicentre	**N:** 146<br>**Postmen n (%):** 76 (52)<br>**Ovarian cancer n (%):** 19 (13)<br>**Borderline n (%):** 7 (5)<br>**Age mean:** 52<br>**Separated by menopausal status:** Yes	**Thresholds:**<br>- Premen 8.6, 13.1;<br>- Postmen 27.7<br>**Pre-specified:** Yes
**Huy 2018 Vietnam**	**Study criteria:** Women with sufficient personal information, clinical symptoms, data on serum CA125 and serum HE4 levels, and postoperative pathologic findings<br>**Clinical setting:** Mixed<br>**Prior test:** Not reported<br>**Exclusions:** Unclear borderline cases<br>**Centre:** Single	**N:** 277<br>**Postmen n (%):** 47 (17)<br>**Ovarian cancer n (%):** 30 (11) (EOC only)<br>**Borderline n (%):** Not reported<br>**Age:** Not reported<br>**Separated by menopausal status:** Yes	**Thresholds:**<br>- Premen 7.4;<br>- Postmen 25.3<br>**Pre-specified:** Yes

**Karlsen 2012**<br>**Denmark**	**Study criteria:** Women admitted to surgery for pelvic mass or pelvic pain potentially caused by malignant disease or endometriosis<br>**Clinical setting:** Secondary<br>**Prior test:** Unclear<br>**Exclusions:** Nil<br>**Centre:** Single	**N:** 1218<br>**Postmen n (%):** 621 (51)<br>**Ovarian cancer n (%):** 261 (21)<br>**Borderline n (%):** 79 (6)<br>**Age mean:** Not reported<br>**Separated by menopausal status:** Yes	**Thresholds:**<br>- Premen 7.4; - Postmen 25.3<br>**Pre-specified:** Yes
**Kadija 2012**<br>**Serbia**	**Study criteria:** Women diagnosed with adnexal mass scheduled to undergo surgery<br>**Clinical setting:** Secondary<br>**Prior test:** Unclear<br>**Exclusions:** Nil<br>**Centre:** Single	**N:** 108<br>**Postmen n (%):** 41 (38)<br>**Ovarian cancer n (%):** 24 (22)<br>**Borderline n (%):** 5 (5)<br>**Age mean:** Not reported<br>**Separated by menopausal status:** Yes	**Thresholds:**<br>- Premen 12.5;<br>- Postmen 14.4<br>**Pre-specified:** No
**Kim 2011**<br>**Korea**	**Study criteria:** Women diagnosed with adnexal mass on the first visit to the gyn oncology clinic and underwent surgery<br>**Clinical setting:** Tertiary<br>**Prior test:** Unclear<br>**Exclusions:** Only EOC included<br>**Centre:** Single	**N:** 159<br>**Postmen n (%):** 108 (68)<br>**Ovarian cancer n (%):** 68 (43)<br>**Borderline n (%):** 10 (6)<br>**Age mean:**<br>- Benign: 35.7<br>- Malignant: 51.7<br>**Separated by menopausal status:** **Yes	**Threshold:**<br>- Premen: 7.6<br>**Pre-specified:** Yes
**Kim 2019**<br>**Korea**	**Study criteria:** Patients with suspected gynaecological disease<br>**Clinical setting:** Tertiary<br>**Prior test:** Unclear<br>**Exclusions:** Unclear; presume BOT excluded as retrospective<br>**Centre:** Single	**N:** 832<br>**Postmen n (%):** 251 (30)<br>**Ovarian cancer n (%):** 70 (8)<br>**Borderline n (%):** Not reported<br>**Median age (IQR):**<br>-Benign: 45.0 (36.0-51.0)<br>-Malignant: 64.0 (50.9-77.0)<br>**Separated by menopausal status:** Yes	**Thresholds:**<br>- Premen 11.4<br>- Postmen 29.9<br>**Pre-specified:** Yes

**Krascsenitis 2016**<br>**Hungary**	**Study criteria:** Women diagnosed with an ovarian tumour of unknown significance admitted for surgery<br>**Clinical setting:** Tertiary<br>**Prior tests:** Not reported<br>**Exclusions:** None reported<br>**Centre:** Single	**N:**162<br>**Postmen n (%):** 102 (63)<br>**Ovarian cancer n (%):** 34 (21)<br>**Borderline n (%):** 11 (7)<br>**Mean age (+/-SD):** 55 (not reported)<br>**Separated by menopausal status:** Yes	**Thresholds:**<br>- Premen 11.4<br>- Postmen 29.9<br>**Pre-specified:** Yes
**Li 2016**<br>**China**	**Study criteria:** Women diagnosed with gynaecologic diseases by US, CT scan, PET-CT scan or MRI<br>**Clinical setting:** Unclear<br>**Prior test:** Not reported<br>**Exclusions:** None reported<br>**Centre:** Single	**N:** 916<br>**Postmen n (%):** 172 (19)<br>**Ovarian cancer n (%):** 190<br>**Borderline n (%):** Not reported<br>**Median age:** 50 (range 18 to 82)<br>**Separated by menopausal status:** Yes	**Thresholds:**<br>- Premen 7.4<br>- Postmen 25.3<br>**Pre-specified:** Yes
**Liest 2017**<br>**Sweden**	**Study criteria:** Women with a pelvic mass of probable ovarian origin and scheduled for surgery<br>**Clinical setting:** Tertiary<br>**Prior tests:** Preoperative US<br>**Exclusions:** None reported<br>**Centre:** Multicentre	**N:** 784<br>**Postmen n (%):** 117 (81)<br>**Ovarian cancer n (%):** 144 (18) (EOC+ borderline)<br>**Borderline n (%):** Not reported<br>**Mean age (+/-SD):** Not reported<br><br>**Separated by menopausal status:** Yes	**Thresholds:**<br>- Premen 11<br>- Postmen 25<br>**Pre-specified:** Yes
**Lycke 2018**<br>**Sweden**	**Study criteria:** Women planned for a surgical procedure for a symptomatic/suspected malignant ovarian cyst or pelvic tumour<br>**Clinical setting:** Mixed<br>**Prior tests:** Unclear but assume history and examination, and US from patient selection<br>**Exclusions:** None reported<br>**Centre:** Multicentre	**N:** 638<br>**Postmen n (%):** 348 (55)<br>**Ovarian cancer n (%):** 162 (25) (EOC only)<br>**Borderline n (%):** 31 (5)<br>**Mean age (+/-SD):**<br>-Benign 50.76 (not reported)<br>-BOT: 55.58 (not reported)<br>-EOC: 62.67 (not reported)<br>**Separated by menopausal status:** Yes	**Thresholds:**<br>- Premen 11.4<br>- Postmen 29.9<br>**Pre-specified:** Yes

**Melo 2018 Portugal**	**Study criteria:** Women with adnexal neoplasia submitted to surgical treatment, with a histological diagnosis and in which ROMA had been determined<br>**Clinical setting:** Tertiary<br>**Prior test:** Unclear<br>**Exclusions:** None reported but age group unclear<br>**Centre:** Single	**N:** 247<br>**Postmen n (%):** 92 (37)<br>**Ovarian cancer n (%):** 34 (14)<br>**Borderline n (%): 7 (3)**<br>**Age:** Not reported<br>**Separated by menopausal status:** Yes	**Thresholds:**<br>- Premen 7.4<br>- Postmen 25.3<br>**Pre-specified:** Yes
**Molina 2011 Spain**	**Study criteria:** Not reported<br>**Clinical setting:** Unclear<br>**Prior test:** Unclear<br>**Exclusions:** Nil<br>**Centre:** Single	**N:** 396<br>**Postmen n (%):** 143 (36)<br>**Ovarian cancer n (%):** 111 (28)<br>**Borderline n (%):** Not reported<br>**Age mean:** Not reported<br>**Separated by menopausal status:** Yes	**Thresholds:**<br>- Premen 13.1;<br>- Postmen 27.7<br>**Pre-specified:** Yes
**Montagnana 2011 Italy**	**Study criteria:** women with pelvic mass scheduled to have radical surgery<br>**Clinical setting:** Secondary<br>**Prior test:** Unclear<br>**Exclusions:** only EOC included<br>**Centre:** Single	**N:** 104<br>**Postmen n (%):** 53 (51)<br>**Ovarian cancer n (%):** 55 (53)<br>**Borderline n (%):** Excluded<br>**Age mean:**<br>**-** Malignant: 56.9<br>- Benign: 42<br>**Separated by menopausal status:** Yes	**Thresholds:**<br>- Premen 12.5;<br>- Postmen 14.4<br>**Pre-specified:** Yes
**Moore 2009 USA**	**Study criteria:** Women with ovarian cyst scheduled to undergo surgery<br>**Clinical setting:** Unclear<br>**Prior test:** Unclear<br>**Exclusions:** Nil<br>**Centre:** Multicentre	**N:** 513<br>**Postmen n (%):** 150 (29)<br>**Ovarian cancer n (%):** 143 (28)<br>**Borderline n (%):** 22 (4)<br>**Age mean:** 54<br>**Separated by menopausal status:** Yes	**Thresholds:**<br>- Premen 13.1;<br>- Postmen 27.7<br>**Pre-specified:** Yes
**Moore 2011 USA**	**Study criteria:** Women with ovarian cyst scheduled to undergo surgery<br>**Clinical setting:** Mixed<br>**Prior test:** Unclear<br>**Exclusions:** Nil<br>**Centre:** Multicentre	**N:** 472<br>**Postmen n (%):** 217 (46)<br>**Ovarian cancer n (%):** 68 (14)<br>**Borderline n (%):** 19 (4)<br>**Age mean:** 50.3	**Thresholds:**<br>- Premen 13.1;<br>- Postmen 27.7<br>**Pre-specified:** Yes

		**Separated by menopausal status:** Yes	
**Nikolova 2016 Macedonia**	**Study criteria:** Premenopausal women to have an ultrasonography scan confirming an ovarian cyst/mass and to undergo surgery<br>**Clinical setting:** Tertiary<br>**Prior test:** Unclear<br>**Exclusions**: Post menopausal women excluded<br>**Centre:** Single	**N:** 105 (analysed)<br>**Postmen n (%):** 0<br>**Ovarian cancer n (%):** 11 (10%) (EOC only)<br>**Borderline n (%):** Not reported<br>**Mean age (+/-SD):**<br>-Malignant: 42.46 (8.21)<br>-Benign: 36.90 (10.12)<br>**Separated by menopausal status:** Only premenopausal women included	**Thresholds:**<br>- Premen 7.4<br>**Pre-specified: Yes**
**Novotny 2012 Czech Republic**	**Study criteria:** Women with pelvic abnormalities<br>**Clinical setting:** Secondary<br>**Prior test:** Unclear<br>**Exclusions:** premenopausal women excluded<br>**Centre:** Single	**N:** 256<br>**Postmen n (%):** 256 (100)<br>**Ovarian cancer n (%):** 21 (8)<br>**Borderline n (%):** Not reported<br>**Age mean:**<br>- Benign: 65.28<br>- Malignant: 64.37<br>**Separated by menopausal status:** Yes	**Thresholds:**<br>- Postmen 26.3<br>**Pre-specified:** No
**Ortiz-Munoz 2014 Spain**	**Study criteria:** Women with gynaecological symptoms, diagnosed with primary ovarian cancer<br>**Clinical setting:** Tertiary<br>**Prior test:** Symptoms<br>**Exclusions:** Nil<br>**Centre:** Single	**N:** 148<br>**Postmen n (%):** 104 (70)<br>**Ovarian cancer n (%):** 29 (20)<br>**Borderline n (%):** Not reported<br>**Age mean:** not reported<br>**Separated by menopausal status:** **Yes	**Thresholds:**<br>- Premen 11.4;<br>- Postmen 29.9<br>**Pre-specified:** Yes
**Park 2019 Korea**	**Study criteria:** Women for whom gynaecologists had requested HE4, CA 125, and ROMA tests to evaluate a pelvic mass<br>**Clinical setting:** Secondary<br>**Prior test:** US, CT or MRI<br>**Exclusions:** 2 cases of non-EOC excluded from analysis<br>**Centre:** Single	**N:** 433 (biopsy 309; follow up 134)<br>**Postmen n (%):**<br>-Biopsy: 81 (26)<br>-Follow up: 37 (28)<br>**Ovarian cancer n (%):** 18 (4)<br>**Borderline n (%):** 15 (3)<br>**Median age:** (check)<br>Benign: 43.0 (21.0)<br>Malignant: 52.3 (6.1)<br>BOT: 47.8 (12.9)<br>**Separated by menopausal status:** Yes	**Thresholds:**<br>- Premen 7.4<br>- Postmen 25.3<br>**Pre-specified:** Yes

**Partheen 2011a**<br>**Sweden**	**Study criteria:** Women with complex cystic mass and suspicious of malignancy undergoing surgery<br>**Clinical setting:** Tertiary<br>**Prior test:** Unclear<br>**Exclusions:** Solid and unilocular mass were excluded<br>**Centre:** Single	**N:** 374<br>**Postmen n (%):** 276 (74)<br>**Ovarian cancer n (%):** 108 (29)<br>**Borderline n (%):** 45 (12)<br>**Age mean:** Not reported<br>**Separated by menopausal status:** **Yes	**Thresholds:**<br>- Premen 17.3<br>- Postmen 26.0<br>**Pre-specified:** Yes
**Prskalo 2019**<br>**Croatia**	**Study criteria:** Women with suspected adnexal mass on a transvaginal US scheduled for elective surgery<br>**Clinical setting:** Mixed<br>**Prior test:** Unclear<br>**Exclusions:** None reported<br>**Centre:** Single	**N:** 159<br>**Postmen n (%):** 102 (64)<br>**Ovarian cancer n (%):** 43 (27)<br>**Borderline n (%):** 11 (7)<br>**Mean age (+/- SD):**<br>-Pre 36.9 (8.9)<br>-Post 60.2 (9.6)<br>**Separated by menopausal status:** Yes	**Thresholds:**<br>- Premen 11.7<br>- Postmen 29.9<br>**Pre-specified:** Yes
**Richards 2015**<br>**Australia**	**Study criteria:** Women who were undergoing surgery for a complex pelvic mass, presumed to be arising from the ovary<br>**Clinical setting:** Mixed<br>**Prior tests:** Unclear<br>**Exclusions:** None reported<br>**Centre:** Single	**N:** 50<br>**Postmen n (%):** 29 (58)<br>**Ovarian cancer n (%):** 16 (32) (EOC only)<br>**Borderline n (%):** 4 (8)<br>**Median age:** 60<br>**Separated by menopausal status:** Yes	**Thresholds:**<br>- Premen 7.4<br>- Postmen 25.3<br>**Pre-specified:** Yes
**Romagnolo 2016**<br>**Italy**	**Study criteria:** Women referred to gynaecological oncologist with a suspicious pelvic mass requiring surgery<br>**Clinical setting:** Tertiary<br>**Prior test:** Pelvic masses were confirmed by US prior to inclusion<br>**Exclusions:** Non EOC<br>**Centre:** Multicentre	**N:** 387<br>**Postmen n (%):** 148 (38)<br>**Ovarian cancer n (%):** 73 (19) (EOC only)<br>**Borderline n (%):** 15 (3.9)<br>**Mean age (+/- SD):**<br>-Premen: 37.6 (8.6)<br>-Postmen: 63 (9.5)<br>**Separated by menopausal status:** Yes	**Thresholds:**<br>- Premen 13.1<br>- Postmen 27.7<br>**Pre-specified:** Yes
**Salim 2018**<br>**Pakistan**	**Study criteria:** Postmenopausal women with ovarian mass (>2cm) on pelvic ultra sound examination, attending gynaecology clinics, planned for surgical intervention<br>**Clinical setting:** Secondary<br>**Prior test:** Not reported<br>**Exclusions:** Only post-menopausal women included<br>**Centre:** Single	**N:** 260<br>**Postmen n (%):** 260 (100)<br>**Ovarian cancer n (%):** 122 (47)<br>**Borderline n (%):** Not reported<br>**Mean age (+/-SD):** 49.28 (6.26)<br>**Separated by menopausal status:** Only post-menopausal women included	**Thresholds:**<br>- Postmen 27.7<br>**Pre-specified:** Yes

**Shen 2017**<br>**China**	**Study criteria:** Women referred to a participating centre with a pelvic mass or an ovarian cyst and planning to undergo surgery<br>**Clinical setting:** Mixed<br>**Prior test:** Pelvic US, CT, MRI and the medical history (the diagnosis and treatment of pelvic mass and history of renal disease)<br>**Exclusions:** None reported<br>**Centre:** Multicentre	**N:** 684<br>**Postmen n (%):** 174 (25)<br>**Ovarian cancer n (%):** 169 (25) (EOC+BOT)<br>**Borderline n (%):** 18 (3)<br>**Mean age (+/-SD):** 58.8 (8.6)<br>**Separated by menopausal status:** Yes	**Thresholds:**<br>-Premen 7.4<br>- Postmen 25.3<br>**Pre-specified:** Yes
**Stiekema 2014**<br>**Netherlands**	**Study criteria:** Histologically confirmed EOC or benign ovarian disease referred to the institute<br>**Clinical setting:** Tertiary<br>**Prior test:** Unclear<br>**Exclusions:** BOT excluded<br>**Centre:** Single	**N:** 181<br>**Postmen n (%):** 143 (79)<br>**Ovarian cancer n (%):** 147 (81)<br>**Borderline n (%):** Excluded<br>**Age mean:**<br>- Benign: 47<br>- Malignant: 57<br>**Separated by menopausal status:** Yes	**Thresholds:**<br>- Premen 12.9;<br>- Postmen 27.8<br>**Pre-specified:** Yes
**Teh 2018**<br>**Malaysia**	**Study criteria:** Women with pelvic mass(es) suspected of originating in the ovary who had been scheduled for surgery or radiological-guided biopsy<br>**Clinical setting:** Tertiary<br>**Prior test:** Not reported<br>**Exclusions:** Unclear; Low malignant potential tumours were included in the benign tumour group during analysis<br>**Centre:** Single centre	**N:** 129<br>**Postmen n (%):** 27 (21)<br>**Ovarian cancer n (%):** 27 (21)<br>**Borderline n (%):** 10 (8)<br>**Median age:** 37 (27.5–48.5)<br>**Separated by menopausal status:** Yes	**Thresholds:**<br>- Premen 11.4<br>- Postmen 29.9<br>**Pre-specified:** Yes
**Terlikowska 2016**<br>**Poland**	**Study criteria:** Caucasian women surgically treated on account of benign ovarian disease and epithelial cancer according to international treatment guidelines<br>**Clinical setting:** Mixed<br>**Prior test:** Not reported<br>**Exclusions:** Non EOC<br>**Centre:** Multicentre	**N:** 224<br>**Postmen n (%):** 104 (46)<br>**Ovarian cancer n (%):** 96 (43) (EOC only)<br>**Borderline n (%):** Not reported<br>**Median age:**<br>-Premen: 36<br>-Postmen: 63<br>**Separated by menopausal status:** Yes	**Thresholds:**<br>- Premen 11.4<br>- Postmen 29.9<br>**Pre-specified:** Yes

**Van Gorp 2011 (Van Gorp 2012- secondary publication- smaller cohort) Belgium**	**Study criteria:** All patients diagnosed with pelvic mass undergoing surgery<br>**Clinical setting:** Unclear<br>**Prior test:** Unclear<br>**Exclusions:** Ni<br>**Centre:** Single	**N**: 389<br>**Postmen n (%):** 161 (41)<br>**Ovarian cancer n (%):** 161 (41)<br>**Borderline n (%):** Not reported<br>**Age mean:**<br>- Benign: 46.3<br>- Malignant: 57.8<br>**Separated by menopausal status:** Yes	**Thresholds:**<br>- Premen 12.5;<br>- Postmen 14.4<br>**Pre-specified:** Yes
**Xu 2016 China**	**Study criteria:** Women with a pelvic mass (defined as a simple, complex or solid ovarian cyst/pelvic mass) and healthy women from the Physical Examination Centre<br>**Clinical setting:** Mixed<br>**Prior test:** Not reported<br>**Exclusions:** Non-EOC<br>**Centre:** Single	**N:** 566<br>**Postmen n (%):** 159 (28)<br>**Ovarian cancer n (%):** 210 (37) (EOC only)<br>**Borderline n (%):** 45 (8)<br>**Mean age (+/-SD):**<br>-Benign: 42 (not reported)<br>-Malignant: 57 (not reported)<br>**Separated by menopausal status:** Yes	**Thresholds:**<br>- Premen 11.4<br>- Postmen 29.9<br>**Pre-specified:** Yes
**Zhang 2015 China**	**Study criteria:** All women scheduled for surgery, with and without pelvic mass on US<br>**Clinical setting:** Unclear<br>**Prior test:** US; Adnexal lesions reported according to IOTA<br>**Exclusions:** Non EOC excluded<br>**Centre:** Multicentre	**N:** 612<br>**Postmen n (%):** 232 (37)<br>**Ovarian cancer n (%):** 264 (43) (EOC only)<br>**Borderline n (%):** Not reported<br>**Median age (25th centile, 75th centile):**<br>-Benign:<br>Pre menopausal: 41 (35, 46)<br>Post menopausal: 57 (54, 68)<br>-Malignant:<br>Pre menopausal: 43 (38, 47)<br>Post menopausal: 59 (54, 65)<br><br>**Separated by menopausal status:** Yes	**Thresholds:**<br>- Premen 11.4<br>- Postmen 29.9<br>**Pre-specified:** Yes

**Zhang 2019**<br>**China**	**Study criteria:** Women with ovarian tumour<br>**Clinical setting:** Tertiary<br>**Prior test:** Unclear<br>**Exclusions:** Borderline excluded from analysis<br>**Centre:** Single	**N:** 373<br>**Postmen n (%):** 185 (50)<br>**Ovarian cancer n (%):** 181 (48)<br>**Borderline n (%):** 17 (5)<br>**Mean age (+/-SD):** 51 (not reported)<br>**Separated by menopausal status:** Yes	**Thresholds:**<br>- Premen 11.4<br>- Postmen 29.9<br>**Pre-specified:** Yes

Study characteristics : ADNEX

Author Year	Study criteria and setting*	Participants characteristics	Index test threshold
**Meys 2017**<br><br>**Netherlands**	**Study criteria:** Women with adnexal pathology<br>**Clinical setting:** Tertiary<br>**Prior tests:** Not reported<br>**Exclusions:** None reported<br>**Centre:** Single	**N:** 326<br>**Postmen n (%):** 198 (61)<br>**Ovarian cancer n (%):** 115 (35)<br>**Borderline n (%):** 27 (8)<br>**Median age (IQR):**<br>-Benign 53.2 (16.1 to 87.2)<br>-Malignant 67.7 (32.3 to 87)<br>**Separated by menopausal status:** Yes	**Threshold:** 10% post test probability of malignancy<br>**Pre-specified:** Yes
**Szubert 2016 (a)**<br><br>**Poland**	**Study criteria:** Women with a 'need for surgery due to an ovarian tumour'<br>**Clinical setting:** Unclear, probably tertiary<br>**Prior test:** Not reported<br>**Exclusions:** None reported<br>**Centre:** Single	**N:** 204<br>**Postmen n (%):** 66 (54)<br>**Ovarian cancer n (%):** 58 (28)<br>**Borderline n (%):** 12 (6)<br>**Median age:** 46<br>**Separated by menopausal status:** Yes	**Thresholds:** 2000 IOTA criteria 10%<br>**Pre-specified:** Yes

**Szubert 2016 (b)**<br><br>**Spain**	**Study criteria:** Women with a 'need for surgery due to an ovarian tumour'<br>**Clinical setting:** Unclear, probably tertiary<br>**Prior test:** Not reported<br>**Exclusions:** None reported<br>**Centre:** Single	**N:** 128<br>**Postmen n (%):** 52 (42)<br>**Ovarian cancer n (%):** 35 (27)<br>**Borderline n (%):** 4 (3)<br>**Median age:** 47<br>**Separated by menopausal status:** Yes	**Thresholds:** 2000 IOTA criteria 10%<br>**Pre-specified:** Yes
**Van Calster 2014**<br><br>**Europe**	**Study criteria:** Women presenting with adnexal mass on US and selected for surgery<br>**Clinical setting:** Mixed secondary and tertiary care<br>**Prior tests:** Not reported<br>**Exclusions:** None reported<br>**Centre**: Multicentre	**N:** 2403<br>**Postmen n (%):** 1049 (43.7)**<br>**Malignant n (%):** 827 (34.4)<br>**Borderline n (%):** 153 (6.4)<br>**Age:** Not reported<br>**Separated by menopausal status:** Yes**	**Threshold:** 3, 5, 10 and 15% post test probability of malignancy<br>**Pre-specified threshold:** Yes
