# Supplementary material for: Diagnostic Models Combining Clinical Information, Ultrasound and Biochemical Markers for Ovarian Cancer: Cochrane Systematic Review and Meta-Analysis
Source: Cancers (Basel). 2022 Jul 26;14(15):3621. doi: 10.3390/cancers14153621 (PMC9332683; doi:10.3390/cancers14153621)
Supplement: Supplementary file 1 [file cancers-14-03621-s001.zip › Supplementary File S4 Pooled estimates test accuracy at thresholds reported in included studies.pdf]

**Supplementary File S4 : Pooled sensitivity and specificity of RMI, ROMA and ADNEX at thresholds reported in included studies**					
**Score, threshold and menopause status**	**Studies**	**Participants**	**OC cases**	**Pooled sensitivity % (95% CI)**	**Pooled specificity % (95% CI)**
**ROMA**					
7.4 (pre)	10	3051	342	80.7 (69.6, 88.5)	80.5 (73.8, 85.9)
25.3 (post)	9	1386	603	86.8 (77.9, 92.5)	87.6 (80.2, 92.6)
11.4 (pre)	11	2281	445	80.9 (71.0, 88.0)	84.1 (81.2, 86.7)
29.9 (post)	12	1797	851	91.6 (84.2, 95.7)	86.3 (80.1, 90.7)
12.5 (pre)	3	302	68	63.5 (51.0, 74.4)	89.3 (80.8, 94.3)
14.4 (post)	3	299	177	88.0 (80.6, 92.8)	68.3 (57.4, 77.4)
13.1 (pre)	8	1353	158	75.2 (67.0, 81.9)	84.0 (78.4, 88.3)
27.7 (post)	9	1265	556	90.5 (86.2, 93.6)	81.1 (75.7, 85.5)
7.4 ±2 (pre)	12	3223	378	80.6 (71.5, 87.3)	81.7 (75.7, 86.5)
25.3 ±2 (post)	15	2599	1049	87.2 (81.7, 91.3)	86.0 (80.3, 90.3)
13.1 ±2 (pre)	27	4463	825	77.8 (72.5, 82.4)	84.3 (81.3, 86.8)
27.7 ±2 (post)	13	2002	852	90.4 (87.4, 92.7)	81.3 (76.9, 85.0)
**RMI 1**					
200 (pre)	17	5233	851	57.1 (50.6, 63.4)	92.5 (90.0, 94.4)
200 (post)	17	4369	1664	78.7 (74.3, 82.5)	85.5 (81.3, 88.9)
Difference in sensitivity & specificity (threshold 200) pre-menopausal versus post-menopausal				21.6 (13.9, 29.2), p<0.0001	-6.9 (-11.3, -2.6), p=0.002
250 (pre)	2	461	42	59.5 (44.3, 73.1)	88.1 (84.6, 90.8)
250 (post)	2	220	97	82.5 (73.6, 88.8)	79.7 (71.6, 85.9)
Difference in sensitivity & specificity (threshold 250) pre-menopausal versus post-menopausal				23.0 (6.3, 39.6), p=0.007	-8.4 (-16.2, -0.6), p=0.034
**ADNEX D+**					
3 (pre)	1	1354	378	97.9 (95.9, 99.1)	56.6 (53.4, 59.7)
3 (post)	1	1049	602	99.5 (98.6, 99.9)	25.1 (21.1, 29.3)
5 (pre)	1	1354	378	97.6 (95.5, 98.9)	69.5 (66.5, 72.3)
5 (post)	1	1049	602	98.8 (97.6, 99.5)	37.4 (32.9, 42.0)
10 (pre)	4	1696	455	94.9 (92.5, 96.6)	78.2 (75.8, 80.4)
10 (post)	4	1365	749	97.6 (96.2, 98.5)	55.2 (51.2, 59.1)
Difference in sensitivity & specificity (threshold 10% post test probability OC) pre-menopausal versus post-menopausal				2.7 (0.4, 4.9), p=0.023	-23.0 (-27.5, -18.4), p<0.0001
15 (pre)	1	1354	378	90.5 (87.1, 93.2)	83.4 (80.9, 85.7)
15 (post)	1	1049	602	96.5 (94.7, 97.8)	63.5 (58.9, 68.0)
