# Supplementary material for: Diagnostic Models Combining Clinical Information, Ultrasound and Biochemical Markers for Ovarian Cancer: Cochrane Systematic Review and Meta-Analysis
Source: Cancers (Basel). 2022 Jul 26;14(15):3621. doi: 10.3390/cancers14153621 (PMC9332683; doi:10.3390/cancers14153621)
Supplement: Supplementary file 1 [file cancers-14-03621-s001.zip › Supplementary File S5 sensitivity analysis borderline tumours.pdf]

Supplementary File S5

Sensitivity analysis: Sensitivity at fixed specificities of 80% and 90% for RMI I and ROMA (all thresholds) for studies grouping borderline ovarian tumours with malignant for the estimation of test accuracy (BOT=1) compared to studies where borderline tumours were excluded or where their management for the estimation of test accuracy was not clear (BOT=2/3)

Test	Studies	Participants	OC Cases	Diagnostic odds ratio (95% CI)	Relative diagnostic odds ratio (95% CI)	p-value	Sensitivity at fixed specificity of 80%		Sensitivity at fixed specificity of 90%	
							Sensitivity (95% CI)	Difference from BOT=1 (95% CI)	Sensitivity (95% CI)	Difference from BOT=1 (95% CI)
**Pre-menopausal**										
RMI 1 200/250										
BOT=1	16	4861	801	11.7 (5.3, 25.9)	-		74.9 (59.6, 85.8)	-	62.2 (53.1, 70.5)	-
BOT=2/3	3	833	92	11.5 (4.2, 31.6)	0.98 (0.37, 2.60)	0.9699	74.6 (55.0, 87.6)	-0.3 (-16.1, 15.5)	61.8 (43.3, 77.4)	-0.4 (-20.1, 19.4)
ROMA mixed										
BOT=1	15	2737	363	13.9 (9.0, 21.7)	-		77.6 (69.1, 84.3)	-	59.2 (47.0, 70.3)	-
BOT=2/3	23	4879	835	22.3 (15.9, 31.3)	1.60 (0.94, 2.74)	0.0837	84.9 (79.7, 89.0)	7.4 (-1.2, 15.9)	70.2 (60.3, 78.6)	11.1 (-1.3, 23.5)
**Post-menopausal**										
ROMA mixed										
BOT=1	15	2289	882	27.4 (18.6, 40.4)	-		87.7 (82.3, 91.7)	-	72.4 (59.6, 82.4)	-
BOT=2/3	25	3810	1864	56.3 (40.5, 78.1)	2.06 (1.24, 3.40)	0.0062	94.1 (91.3, 96.0)	6.4 (1.2, 11.5)	85.4 (79.6, 89.8)	13.0 (1.9, 24.0)
